# Case Report—Extra-Uterine Superfetation

**Published:** 1894-09

**Authors:** R. R. Kime

**Affiliations:** Atlanta, Ga.


### CASE REPORT—EXTRA-UTERINE SUPERFETATION.

By R. R. KIME, M.D.,
Atlanta, Ga.

Case referred to me by Dr. Hall, September 28, 1893. Jennie Fuller, colored, married, age thirty years, had two children, youngest four years of age.

In January, 1890, had what she thought to be a miscarriage, believing herself to be pregnant some two or three months. At the time suffered severe pain, free hemorrhage, and passed what was thought to be material the result of a normal pregnancy.

Missing her flow again in December, 1892, with indications of pregnancy, but suffering so much pain, later a physician was called, diagnosing extrauterine pregnancy. Improved to some extent, but suffered occasional attacks of pain with no discharge of blood until the last of July, 1892, at which time there was considerable discharge of blood, and *severe attacks of pain;* since, at intervals of eight to ten days, discharges of blood occur, accompanied with considerable pain. Fetal movements were active and vigorous prior to attack of pain and hemorrhage in July; since then movements ceased, and breasts became flabby. At first visit, pulse 124, temperature 102.5° F., bowels distended, tympanitic, with evidences of infection. Examination revealed a large cyst containing fetus and operation advised.

September 30th, Drs. Crow, Davis, Gregory, Hall and Kime, Jr., saw patient, and concurred in diagnosis. Operation again advised, but refused, and patient died eight days after.

Post Mortem.—Median incision revealed a large cyst extending from pubes above umbilicus two or three inches; cyst cavity reached without entering abdominal cavity. It contained bloody, purulent, putrid fluid, a fetus weighing about six or seven pounds partially decomposed, with placenta *in situ.* Buttocks of fetus in anterior-superior portion of cyst, extending a little to left of median line, dorsum of fetus to the right, vertex R. O. posterior position, sutures felt plainly through rectum, partial decomposition of fetus, cyst wall inflamed and adherent to abdominal walls, intestines, bladder, uterus, etc. Fetus removed, placenta attached to fundus, and right side of uterus and cyst wall involving bladder, promontory of sacrum and ascending colon filled with coagulated blood not decomposed.

In removing placenta a second cyst was encountered in region of bladder and anterior to where uterus should have been, cyst posterior to pubes extending a little above and a little to left of median line.

In examination of patient this cyst was mistaken for the uterus.

Opening cyst found it contained a macerated fetus, and from its state of decomposition, maceration, previous development gave evidences of having been encysted some months.

REMARKS.—From position of large cyst and fetus it must have escaped from right tube by extraperitoneal rupture and developed to near eight months of gestation before its death, about the first of August, 1893. The smaller fetus was the result of pregnancy occurring in the left tube, giving indications of a miscarriage in January, 1890, nearly two years previous to last impregnation. The first tubal pregnancy in left side also ruptured extraperitoneally and died later. I present this brief report and history of this case with specimens in order to place on record a unique unusual case. So far as I know there are but few similar cases on record, and they principally of ancient origin.

I have a case on hand now which I am watching with considerable interest, that presents a strong probability of contemporaneous uterine and extrauterine pregnancy.

---

### AN APPLICATION FOR ENLARGED GLANDS.

℞ Iodoformi,

Bals. Peruviani, of each ............................................. ʒ i.

Collodii ........................................................ ℥ i.

M. To be painted over the swellings every night.—*Ex.*

